# Supplementary material for: RCy3: Network biology using Cytoscape from within R
Source: F1000Res. 2019 Dec 4;8:1774. Originally published 2019 Oct 18. [Version 3] doi: 10.12688/f1000research.20887.3 (PMC6880260; doi:10.12688/f1000research.20887.3)
Supplement: Peer review history

# Peer review: Reviewer response for version 2

**DOI:** 10.5256/f1000research.23645.r56320

**Published:** 2019-11-28

**Authors:** Ludwig Geistlinger

**Affiliations:** 1 Department of Epidemiology and Biostatistics, City University of New York (CUNY), Graduate School of Public Health and Health Policy, New York, NY, USA

Gustavsen and colleagues present a well-written software article that describes usage of RCy3, a R/Bioconductor package for using Cytoscape from within R.

 The package is very well documented in both analysis vignettes on the Bioconductor landing page of the package and in the article itself.

 The implemented functionality covers a range of useful features including network import/export, network layout, and integration with network and enrichment analysis packages.

 I have 3 minor comments:

- Table 2 is supposed to show the "Top 30 network-related Bioconductor packages ordered by rank" but misses the EnrichmentBrowser package (http://bioconductor.org/packages/EnrichmentBrowser) which is (and was in Sep 2019) highly ranked in the various corresponding BiocViews:

Rank 2: https://bioconductor.org/packages/release/BiocViews.html#___NetworkEnrichment

 Rank 10: https://bioconductor.org/packages/release/BiocViews.html#___Network

 Rank 13: https://bioconductor.org/packages/release/BiocViews.html#___GraphAndNetwork

 The relevant functions EnrichmentBrowser::compileGRN (compiles a gene regulatory network in a simple 3 column format regulator-target-effectType), EnrichmentBrowser::nbea (network-based enrichment analysis working on such GRNs), and EnrichmentBrowser::ggeaGraph (constructs a graphNEL object that annotates differential expression and edge consistency) produce network-representations that are compatible with the formats supported by RCy3. I tested that.

- The lines

gal80Rexp.min <- min(data$gal80Rexp, na.rm = T)

 gal80Rexp.max <- max(data$gal80Rexp, na.rm = T)

 should be replaced with

 gal80Rexp.min <- min(data$gal80Rexp, na.rm = TRUE)

 gal80Rexp.max <- max(data$gal80Rexp, na.rm = TRUE)

 consistent with the remainder of the article and good R style.

- The lines

# Run EnrichmentMap build command

 em_command <- paste('enrichmentmap build analysisType="generic"',

 "gmtFile=", paste(getwd(), gmt.file, sep="/"),

 "pvalue=", 1,

 "qvalue=", 1,

 "similaritycutoff=",0.25,

 "coefficients=","JACCARD")

 can be simplified to

 # Run EnrichmentMap build command

 em_command <- paste('enrichmentmap build analysisType="generic"',

 paste0("gmtFile=", file.path(getwd(), gmt.file)),

 "pvalue=1",

 "qvalue=1",

 "similaritycutoff=0.25",

 "coefficients=JACCARD")

I confirm that I have read this submission and believe that I have an appropriate level of expertise to confirm that it is of an acceptable scientific standard.

# Response

**Published:** 2019-12-01

**Authors:** Alexander Pico

**Affiliations:** Institute of Data Science and Biotechnology, Gladstone Institutes, San Francisco, CA, USA

Thanks for the review and code style suggestions. Sorry we missed your package! I'll look into adding it to the table.

# Peer review: Reviewer response for version 2

**DOI:** 10.5256/f1000research.23645.r57133

**Published:** 2019-11-27

**Authors:** James Denvir

**Affiliations:** 1 Department of Biomedical Sciences, Joan C. Edwards School of Medicine, Marshall University, Huntington, WV, USA

I have no further comments.

I confirm that I have read this submission and believe that I have an appropriate level of expertise to confirm that it is of an acceptable scientific standard.

# Peer review: Reviewer response for version 1

**DOI:** 10.5256/f1000research.22981.r55401

**Published:** 2019-11-21

**Authors:** Krithika Bhuvaneshwar

**Affiliations:** 1 Innovation Center for Biomedical Informatics, Georgetown University Medical Center, Washington, DC, USA

This is a review of the manuscript titled "RCy3: Network biology using Cytoscape from within R". RCy3 is a Bioconductor package that communicates with Cytoscape via its REST API to build and visualize networks. This manuscript is about a re-designed/updated RCy3 package and its function and implementation.

 My comments are below:

- The authors say "We have re-written every function". Is it backward compatible? Should existing users of the older version of the package have to make changes in their code?
- The authors say "Over 100 new functions have been added, including dozens of helper functions specifically for intuitive data overlay operations". This is a great achievement. In addition to this statement, perhaps it would be helpful for users to summarize/compare what the main improvements are in this newer version?
- Does this newer version include more or better (or the same) interoperability with other major packages that use RCy3? For example, exporting and importing to/from Ndex. From the example code listed in the paper, it seems there is no change in the way the interoperability works?

Minor comment:

 It is helpful to call the package RCy3 2.0 or something to note the version change? Would keeping the same name be confusing for users?

 Overall great work! RCy3 is one of the major packages that work with Cytoscape so very important for the R/BioC Community. Not all authors of Bioconductor and R packages take effort to update their packages, so I commend this effort. I have used RCy3 in the past and am excited to try out this newest version of the package.

I confirm that I have read this submission and believe that I have an appropriate level of expertise to confirm that it is of an acceptable scientific standard.

# Response

**Published:** 2019-11-21

**Authors:** Alexander Pico

**Affiliations:** Institute of Data Science and Biotechnology, Gladstone Institutes, San Francisco, CA, USA

Thank you for your careful review and questions. The package is *not* backward compatible. We in fact initially submitted it as a completely new package, but were encouraged by Bioconductor reviewers to coordinate with the original RCy3 authors and submit it as a new version of the same package, which we did. The version number is kept separate from the package name per Bioconductor and semantic versioning conventions, so we maintained the original package name. There is a vignette dedicated to upgrading scripts from the original to version 2: ( https://bioconductor.org/packages/release/bioc/vignettes/RCy3/inst/doc/Upgrading-existing-scripts.html), which highlights the major differences and improvements.

 The original version focused on graphNEL objects, so interoperability with other graphNEL-based packages perhaps hasn’t changed. But I just now double checked the old code base and I can’t find any NDEx, igraph, data frame or adjacency matrix related functions for network interoperability in the original version.

# Peer review: Reviewer response for version 1

**DOI:** 10.5256/f1000research.22981.r55403

**Published:** 2019-10-31

**Authors:** James Denvir

**Affiliations:** 1 Department of Biomedical Sciences, Joan C. Edwards School of Medicine, Marshall University, Huntington, WV, USA

This article presents an R package, RCy3, designed to control and automate the network visualization software application Cytoscape. The article describes both a low-level ("generic") and a high-level ("specific") API and presents use-cases with some code samples.

 Overall this appears to be an API with great potential utility for the bioinformatics community, and the article presents the package clearly and with several useful and informative examples. 

 I have a couple of minor comments and suggestions, which I hope will serve to improve the article.

 The abstract mentions the potential for RCy3 to deliver reproducible workflows. In my opinion, reproducibility is among the most important benefits of code/script-based workflows over workflows performed using "point and click" GUI-based applications. However, the article does not elaborate on this outside of the abstract. A couple of sentences in the discussion describing the potential for RCy3 to enhance reproducibility would be worthwhile.

 The article mentions that the Cytoscape App store currently lists over 40 "RCy3-supported apps". A quick note on what is needed for a third party app to be "RCy3-supported" might be pertinent here. This may be already sufficiently covered in existing publications (e.g. reference 6), in which case a simple note to that effect would suffice.

 The code block for the section "Building maps of enrichment analysis results" is not quite as clear as the other code blocks in the article. A comment indicating that commandsAPI() opens a web browser page (externally; at least in my environment) and comments after the print() statements showing the expected output, as in the other blocks of code, would be helpful. The uses of file.path and paste(... sep='/') are a little confusing here (to be honest, I was surprised to find these actually worked on Windows systems); brief comments added to the code might aid readability here.

I confirm that I have read this submission and believe that I have an appropriate level of expertise to confirm that it is of an acceptable scientific standard.

# Response

**Published:** 2019-11-21

**Authors:** Alexander Pico

**Affiliations:** Institute of Data Science and Biotechnology, Gladstone Institutes, San Francisco, CA, USA

Thank you for your careful review and suggestions. We share your opinion regarding the importance of reproducibility and will add a sentence accordingly to the Discussion section. The support of automation by Cytoscape apps is indeed a major topic of reference 6; we will note that in the text as well. Regarding the code block, we will likely remove the commandsAPI() call to not surprise/distract users with a browser window opening. The use of file.path is simply to avoid a really long string, i.e., longer than the column width of this publication. The use of forward slash is in paths is a generic solution to avoid platform-specific conventions and avoid having to escape backslashes. Neither of these last two “tricks” are at all specific to RCy3, so we might just leave those as-is.
